# Bilateral Cortical-sparing Adrenalectomy for the Treatment of Bilateral Aldosterone-producing Adenomas

**DOI:** 10.1210/jcemcr/luad144

**Published:** 2023-12-07

**Authors:** Kazutaka Nanba, Hiroki Kaneko, Mutsuki Mishina, Tetsuya Tagami

**Affiliations:** Department of Endocrinology and Metabolism, National Hospital Organization Kyoto Medical Center, 612-8555 Kyoto, Japan; Department of Endocrinology, Metabolism, and Hypertension Research, Clinical Research Institute, National Hospital Organization Kyoto Medical Center, 612-8555 Kyoto, Japan; Department of Endocrinology and Metabolism, National Hospital Organization Kyoto Medical Center, 612-8555 Kyoto, Japan; Department of Medicine and Bioregulatory Science, Graduate School of Medical Sciences, Kyushu University, 812-8582 Fukuoka, Japan; Department of Urology, National Hospital Organization Kyoto Medical Center, 612-8555 Kyoto, Japan; Department of Endocrinology and Metabolism, National Hospital Organization Kyoto Medical Center, 612-8555 Kyoto, Japan; Department of Endocrinology, Metabolism, and Hypertension Research, Clinical Research Institute, National Hospital Organization Kyoto Medical Center, 612-8555 Kyoto, Japan

**Keywords:** primary aldosteronism, bilateral aldosterone-producing adenomas, CYP11B2, cortical-sparing adrenalectomy

## Abstract

Aldosterone-producing adenoma (APA) is 1 of the major subtypes of primary aldosteronism (PA). Although most APA occurs unilaterally, bilateral APAs have rarely been documented. Because of its rarity, optimal management of patients with bilateral APAs has not been established. Here, we report a case of bilateral APAs that was successfully treated with simultaneous bilateral cortical-sparing surgery. A 44-year-old Japanese woman was referred to us for the evaluation of PA. She had typical clinical characteristics of PA, including hypertension, hypokalemia, and high plasma aldosterone concentration with suppressed renin. She was diagnosed as having PA based on the results of confirmatory testing. Computed tomography revealed bilateral adrenal nodules with diameters of 17 and 10 mm on the right and left adrenal gland, respectively. Adrenal venous sampling indicated excess aldosterone production from bilateral adrenal lesions. She underwent simultaneous bilateral laparoscopic partial adrenalectomy that normalized her blood pressure and serum potassium levels. Aldosterone synthase immunohistochemistry on the resected adrenal tumor tissue confirmed the diagnosis of bilateral APAs. Long-term postsurgical follow-up data suggest cure of the disease without the need for glucocorticoid replacement therapy. Bilateral cortical-sparing adrenalectomy appears to be a viable treatment option at least for selected patients with bilateral APAs.

## Introduction

Primary aldosteronism (PA) is the most common form of endocrine-related hypertension, characterized by hypertension and often hypokalemia. Because of high cardiovascular morbidity and mortality, appropriate diagnosis and targeted treatment are recommended by clinical practice guidelines [1, 2]. Aldosterone-producing adenoma (APA) and idiopathic hyperaldosteronism (IHA), also known as bilateral adrenal hyperplasia, are the major subtypes of PA. Subtype classification is crucial because treatment approaches are different. Because most APA is a unilateral disease, it can be cured by unilateral adrenalectomy. On the other hand, IHA requires lifelong medical treatment, including mineralocorticoid receptor antagonists.

Rarely, APAs occur in bilateral adrenal glands. Because of its rare incidence, optimal management for bilateral APAs is not standardized. Here, we report a case of bilateral APAs treated by simultaneous bilateral laparoscopic cortical-sparing surgery. The resected tumors were histologically confirmed as APA by aldosterone synthase (CYP11B2) immunohistochemistry (IHC). Long-term postsurgical follow-up data indicate cure of PA with no aid of glucocorticoid replacement therapy.

## Case Presentation

A 44-year-old Japanese woman was referred to us for the work-up of PA. Hypertension was noted 5 to 6 years before presentation. She had no family history of endocrine disorders. At the age of 41 years, antihypertensive medications were administered at a local clinic. At the age of 43 years, she was transported to a hospital because of sudden chest pain. She underwent coronary angiography and was diagnosed as having idiopathic coronary artery dissection. During hospitalization, laboratory testing revealed spontaneous hypokalemia (3.0 mmol/L [3.0 mEq/L]; normal reference range [RR], 3.6-4.9 mmol/L [3.6-4.9 mEq/L]) and high plasma aldosterone concentration (PAC, 987.5 pmol/L [35.6 ng/dL]; RR, 83.2-441.1 pmol/L, [3.0-15.9 ng/dL]) with suppressed plasma renin activity (PRA, 0.3 μg/L/h [0.3 ng/mL/h]; RR, 0.2-2.7 μg/L/h [0.2-2.7 ng/mL/h]).

## Diagnostic Assessment

**Figure 1.**
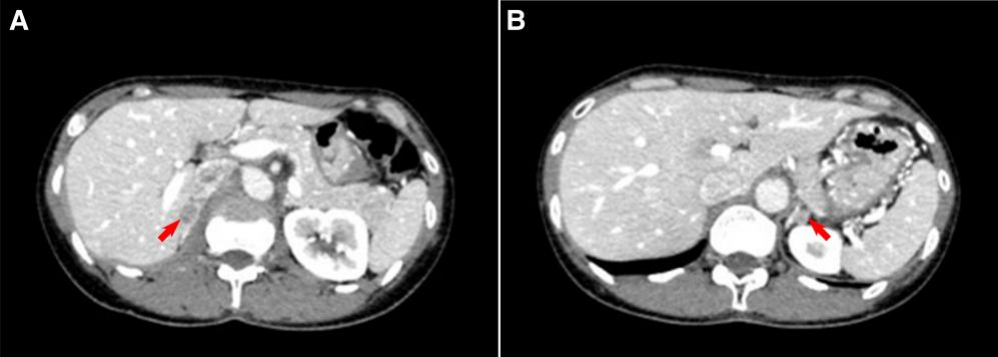
Results of adrenal computed tomography. (A and B) Representative images of contrast-enhanced adrenal CT scan. Red arrows indicate adrenal tumors (A, Right adrenal tumor. B, Left adrenal tumor). CT, computed tomography.

At presentation, her blood pressure was 124/89 mm Hg under treatment with benidipine 4 mg twice daily and azilsartan 20 mg once daily. Her body mass index was 19.5 kg/m^2^. Her kidney function was normal with serum creatinine of 42.4 µmol/L (0.48 mg/dL) (RR, 40.0-65.4 µmol/L [0.35-0.74 mg/dL]). Her hypokalemia remained despite potassium supplementation (32 mEq per day). She underwent confirmatory testing for the diagnosis of PA, including a captopril challenge test and seated saline infusion test. As shown in Table 1, the results of both tests supported the diagnosis of PA [1, 2]. Serum cortisol after overnight 1-mg dexamethasone suppression test was 21.7 nmol/L (0.786 µg/dL) (RR, <49.7 nmol/L [<1.8 µg/dL [2]]), indicating no concomitant cortisol secretion. Computed tomography (CT) revealed bilateral adrenal nodules with the diameters of 17 and 10 mm in the right and left adrenal gland, respectively (Fig. 1A and 1B). Adrenal venous sampling (AVS) with cosyntropin, synthetic 1-24 ACTH stimulation showed lateralized index (the ratio of adrenal venous PAC/cortisol in the dominant side divided by that in the nondominant side) of 2.0 and contralateral ratio of 1.1 (the ratio of PAC/cortisol in the nondominant side divided by that in the inferior vena cava), suggesting excess aldosterone production from both of the adrenal glands (Table 2) [2]. Based on the disease onset at a relatively young age, severity of the disease, a solitary adenoma in each adrenal gland on CT, and the AVS results, the patient was diagnosed as having bilateral APA rather than IHA.

**Table 1.** Results of confirmatory testing

	Values
Captopril challenge test^a^	
Baseline PAC	33.3 ng/dL (923.7 pmol/L)
Baseline PRA	< 0.2 ng/mL/h (< 0.2 µg/L/h)
60-min PAC	35.5 ng/dL (984.8 pmol/L)
60-min PRA	< 0.2 ng/mL/h (< 0.2 µg/L/h)
90-min PAC	34.9 ng/dL (968.1 pmol/L)
90-min PRA	< 0.2 ng/mL/h (< 0.2 µg/L/h)
Seated saline infusion test^b^	
Baseline PAC	52.2 ng/dL (1448.0 pmol/L)
Baseline PRA	< 0.2 ng/mL/h (< 0.2 µg/L/h)
4-h PAC	45.7 ng/dL (1267.7 pmol/L)
4-h PRA	< 0.2 ng/mL/h (< 0.2 µg/L/h)

*Abbreviations:* PAC, plasma aldosterone concentration; PRA, plasma renin activity.

^*a*^Aldosterone to renin ratio [PAC (ng/dL) / PRA (ng/mL/h)]≥20 at 60 or 90 minutes after 50 mg of captopril administration was considered as a positive result (2).

^*b*^PAC>6 ng/dL after 4 h saline infusion was considered as a positive result (1).

**Table 2.** Adrenal venous sampling results

	Right adrenal vein	Left adrenal vein	IVC
PAC	7848.2 ng/dL (217709 pmol/L)	4470.4 ng/dL (124009 pmol/L)	108.8 ng/dL (3018 pmol/L)
Cortisol	569.1 µg/dL (15699 nmol/L)	646.3 µg/dL (17829 nmol/L)	17.2 µg/dL (474 nmol/L)

Adrenal venous sampling was performed with cosyntropin stimulation. PAC-to-cortisol ratio (A/C) using conventional units were 13.8, 6.9, and 6.3 in.right adrenal vein, left adrenal vein, and IVC, respectively.

*Abbreviations:* PAC, plasma aldosterone concentration; IVC, inferior vena cava.

## Treatment

Treatment options were discussed with the patient. She desired surgical cure of PA and underwent simultaneous bilateral laparoscopic partial adrenalectomy. Glucocorticoid replacement was performed during the perioperative period to avoid adrenal insufficiency. PAC on postoperative day 1 was below a detectable limit. There were no major complications after surgery. Cosyntropin stimulation test was performed on postoperative day 6. The cortisol levels of pre- and postcosyntropin stimulation (250 µg) were 187.5 nmol/L (6.80 µg/dL) and 275.0 nmol/L (9.97 µg/dL) at 60 minutes, respectively. Glucocorticoid replacement was discontinued on the same day. She was discharged on postoperative day 9. At discharge, hydrocortisone tablets were prescribed to be taken in case of adrenal insufficiency.

## Outcome and Follow-up

**Figure 2.**
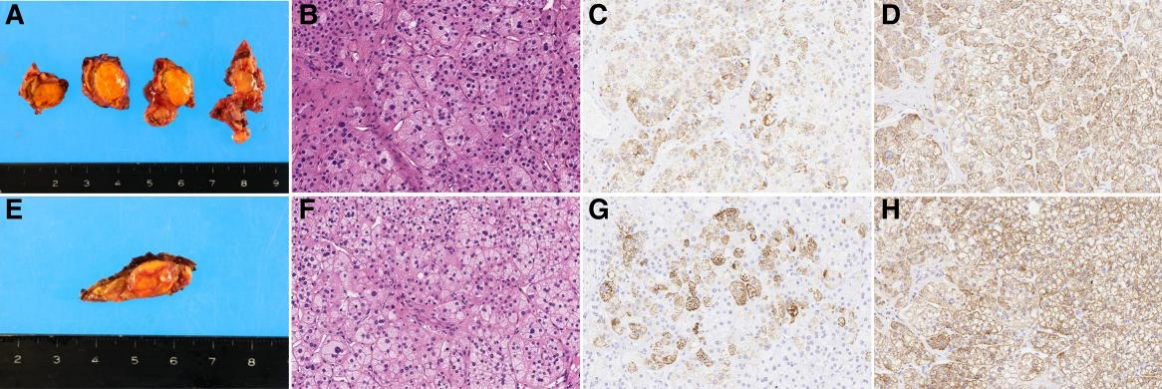
Histologic findings of the resected adrenal tumors. (A-D) Histologic findings of right adrenal tumor. (E-H) Histologic findings of left adrenal tumor. (A and E) Cut surfaces of the resected adrenal tumors. (B and F) Hematoxylin and eosin staining. (C and G) CYP11B2 immunohistochemistry. (D and H) CYP11B1 immunohistochemistry.

The excised adrenal tissue contained 22- and 11-mm tumors in right and left adrenal gland, respectively. The right adrenal tumor showed a yellowish cut surface (Fig. 2A). Microscopically, the tumor was mainly composed of lipid-rich clear cells (Fig. 2B) and was diagnosed as adrenocortical adenoma. By IHC, the right adrenal tumor cells demonstrated immunoreactivity to CYP11B2, a steroidogenic enzyme required for aldosterone biosynthesis (Fig. 2C). Immunoreactivity for 11β-hydroxylase (CYP11B1), a steroidogenic enzyme required for cortisol production, was also observed (Fig. 2D). The left adrenal tumor had similar characteristics to those seen in the right adrenal tumor (ie, yellowish cut surface of the tumor) (Fig. 2E), histologic characteristics of adrenocortical adenoma with lipid-rich clear cells (Fig. 2F), and positive CYP11B2 and CYP11B1 expression (Fig. 2G and 2H). Both adrenal tumors were histologically diagnosed as APAs according to the histopathology of PA (HISTALDO) consensus [3]. No other CYP11B2-expressing lesions, such as aldosterone-producing micronodules (APM), were observed in the adjacent adrenal tissue.

After surgery, the patient’s blood pressure and serum potassium normalized. Aldosterone to renin ratio (ARR; PAC [ng/dL]/PRA [ng/mL/h]) also fell below 20, which is a commonly used cut-off value for the screening of PA in Japanese centers [2]. According to the criteria proposed by the PA surgical outcome (PASO) study [4], complete clinical and biochemical success was achieved during the long-term postsurgical follow-up (Table 3). Cosyntropin stimulation tests were repeated at the follow-up visits 1, 2, and 3 years after surgery. The response to cosyntropin improved over time, as shown in Table 3. No evidence of tumor recurrence in the residual adrenal glands was observed on CT that was performed 2 years after surgery.

**Table 3.** Postoperative follow-up data

	Postoperative follow-up timing			
	1 year	2 years	3 years	4 years
Baseline characteristics				
Blood pressure	117/83 mmHg	109/87 mmHg	102/75 mmHg	124/74 mmHg
Serum creatinine	0.61 mg/dL	0.74 mg/dL	0.72 mg/dL	0.75 mg/dL
	(53.9 µmol/L)	(65.4 µmol/L)	(63.7 µmol/L)	(66.3 µmol/L)
Serum potassium	4.0 mEq/L	3.9 mEq/L	4.1 mEq/L	4.2 mEq/L
	(4.0 mmol/L)	(3.9 mmol/L)	(4.1 mmol/L)	(4.2 mmol/L)
PAC	< 5 ng/dL	8.3 ng/dL	1.8 ng/dL	2.6 ng/dL
	(< 138.7 pmol/L)	(230.2 pmol/L)	(49.9 pmol/L)	(72.1 pmol/L)
PRA	1.3 ng/mL/h	1.8 ng/mL/h	3.2 ng/mL/h	5.6 ng/mL/h
	(1.3 µg/L/h)	(1.8 µg/L/h)	(3.2 µg/L/h)	(5.6 µg/L/h)
ACTH	21.8 pg/mL	NA	20.0 pg/mL	24.0 pg/mL
	(4.8 pmol/L)		(4.4 pmol/L)	(5.3 pmol/L)
Cortisol	5.0 µg/dL	5.9 µg/dL	5.3 µg/dL	5.7 µg/dL
	(137.9 nmol/L)	(162.8 nmol/L)	(146.2 nmol/L)	(157.2 nmol/L)
Cosyntropin stimulation test				
30-min Cortisol	11.7 µg/dL	13.6 µg/dL	13.4 µg/dL	NA
	(322.8 nmol/L)	(375.2 nmol/L)	(369.7 nmol/L)	
60-min Cortisol	14.8 µg/dL	16.4 µg/dL	16.2 µg/dL	NA
	(408.3 nmol/L)	(452.4 nmol/L)	(446.9 nmol/L)	
90-min Cortisol	NA	NA	18.1 µg/dL	NA
			(499.3 nmol/L)	

*Abbreviations:* ACTH, adrenocorticotropic hormone; PAC, plasma aldosterone concentration; PRA, plasma renin activity; NA, not available.

## Discussion

The most common subtype of bilateral PA is sporadic IHA. Other, rarer subtypes of bilateral PA include bilateral APAs and familial hyperaldosteronism. Although the exact prevalence of bilateral APA is not well established, a study by Wu et al [5] determined 7 patients with bilateral APAs in their cohort of 164 patients with APA, resulting in a prevalence of 4.3% of APA. In patients with PA and bilateral adrenal nodules, it is sometimes difficult to differentiate between APA(s) and IHA because an image-identified tumor can be nonfunctioning or other functioning tumors such as cortisol-producing adenoma. The study by Wu et al [5] demonstrated that patients with bilateral APA had lower serum potassium levels, lower PRA, higher PAC, and higher ARR after captopril challenge test compared with those in IHA. The authors suggested that patients with low serum potassium and ARR > 100 (ng/dL per ng/mL/h) after captopril challenge test should be carefully evaluated for bilateral APAs. Consistent with this, our case showed low serum potassium levels and high ARR after captopril challenge test (ARR 60 minutes after 50 mg of captopril administration > 177.5 ng/dL per ng/mL/h). Some researchers recommend the use of segmental AVS for differentiation between bilateral APAs and IHA because it allows evaluation of intra-adrenal hormone production [6]. Segmental AVS can also provide useful information to determine surgical approach. However, the indication of segmental AVS should be carefully assessed because it requires more specialized technique, higher costs, and longer procedure time compared with conventional AVS [6]. Segmental AVS may also increase risks of complications such as adrenal hemorrhage [6]. As alternatives to AVS, functional imaging techniques, especially newly developed selective nuclear imaging modalities such as dexamethasone-suppressed ^11^C-metomidate positron emission tomography-CT [7] and ^68^Ga-pentixafor positron emission tomography-CT [8], may provide useful information to differentiate bilateral APA from IHA. Notably, bilateral APAs (second APA) can be found sequentially after initial unilateral surgery for APA [5, 9], suggesting the importance of long-term follow-up of surgically treated patients with PA.

The HISTALDO consensus demonstrated diverse CYP11B2 expression patterns in adrenals with PA, including APA, aldosterone-producing nodule (APN), APM, multiple APNs, multiple APMs (MAPM), and aldosterone-producing diffuse hyperplasia (APDH) [3]. Among these histologic subtypes, the vast majority of adrenals from young adult patients with PA (aged <35 years) show classical histopathology (APA or APN) [10] associated with a higher rate of postsurgical clinical success compared with that in nonclassical histopathology (multiple APN, MAPM, or APDH) [11]. Although accurate age of the disease onset was unclear, our patient was hypertensive since the age of 38 or 39 years. Likely, the patient had PA at that time because her blood pressure normalized after surgery. Considering the relatively young onset of the disease, florid phenotype of PA, a solitary adenoma in each adrenal gland by CT, and the AVS results, the patient was diagnosed as having bilateral APAs rather than IHA and underwent bilateral partial adrenalectomy. Because small aldosterone-producing lesions such as APM frequently occur, especially in the elderly [12], partial adrenalectomy may leave these lesions in the residual adrenal tissue, potentially contributing to persistent aldosterone excess. However, a recent multicenter retrospective study demonstrated comparable PASO-based clinical and biochemical success rates between minimally invasive partial vs total adrenalectomy for the treatment of unilateral PA [13]. Dedicated prospective studies are needed to determine the potential of partial adrenalectomy for PA.

In a recent international retrospective cohort study, Williams et al [14] performed the assessment of postsurgical outcomes and histologic analysis of resected adrenal glands from surgically treated patients with bilateral PA. In their cohort, 13 patients underwent bilateral adrenalectomy. At 6 to 12 months of postsurgical follow-up, complete clinical and biochemical success rates according to the PASO criteria were 46% (6/13) and 85% (11/13), respectively. Adrenal insufficiency was observed in 31% (4/13). Histologic analysis of 13 pairs of bilaterally resected adrenal glands using CYP11B2 IHC revealed bilateral APA in 5 pairs according to the HISTALDO criteria. The remaining included 3 pairs of bilateral MAPM, 2 pairs of bilateral APDH, and 3 pairs of adrenal glands with different histopathology in each side. When focusing on histologically proven bilateral APAs, complete clinical and biochemical success rates at 6 to 12 months of postsurgical follow-up were 60% (3/5) and 100% (5/5), respectively [14]. Our case showed excellent postsurgical outcomes with complete clinical and biochemical success up to 4 years after surgery.

Several disease-causing somatic mutations has been identified in APA. However, genetic causes of bilateral APA are largely unknown. In a recent study, Kong et al [9] sequenced DNA from sequentially occurred bilateral APA and identified somatic *KCNJ5* c.451G > A and c.451G > C mutations (both resulted in p.G151R) in the left and right APA, respectively. Determination of somatic mutations may provide important information for the development of future diagnostics such as 18-oxocortisol as a potential biomarker for *KCNJ5*-mutated APA that often demonstrates coexpression of CYP11B2 and CYP11B1, as in our case [15]. Considering the bilateral nature of the disease, there may also be a genetic predisposition in patients with bilateral APAs. Further studies are needed.

In conclusion, we report a case of bilateral APAs that were successfully treated by bilateral partial adrenalectomy. The resected tumors were histologically diagnosed as APA by CYP11B2 IHC. Long-term postsurgical follow-up data indicate clinical and biochemical resolution of PA without need for glucocorticoid replacement therapy. Although the indication of bilateral cortical-sparing surgery requires careful preoperative assessment, this surgical approach appears to be a viable treatment option at least for selected patients with bilateral APAs.

## Learning Points

- Aldosterone-producing adenomas can occur bilaterally.
- Bilateral partial adrenalectomy is a viable treatment option at least for selected patients with bilateral aldosterone-producing adenomas.
- Accumulation of evidence is needed to establish optimal management of patients with bilateral aldosterone-producing adenomas.

## Data Availability

Data sharing is not applicable to this article as no datasets were generated or analyzed during the current study.

## Abbreviations

APA: aldosterone-producing adenoma
APDH: aldosterone-producing diffuse hyperplasia
APM: aldosterone-producing micronodule
APN: aldosterone-producing nodule
ARR: aldosterone to renin ratio
AVS: adrenal venous sampling
CT: computed tomography
CYP11B1: 11β-hydroxylase
CYP11B2: aldosterone synthase
IHA: idiopathic hyperaldosteronism
IHC: immunohistochemistry
MAPM: multiple aldosterone-producing micronodule
PA: primary aldosteronism
PAC: plasma aldosterone concentration
PASO: PA surgical outcome study
PRA: plasma renin activity
RR: reference range
